# Database of osmoregulated proteins in mammalian cells

**DOI:** 10.14814/phy2.12180

**Published:** 2014-10-29

**Authors:** Cameron R. Grady, Mark A. Knepper, Maurice B. Burg, Joan D. Ferraris

**Affiliations:** 1 Systems Biology Center, National Heart, Lung and Blood Institute, National Institutes of Health, Bethesda, Maryland, USA

**Keywords:** Database, hypertonicity, phosphorylation, protein abundance

## Abstract

Biological information, even in highly specialized fields, is increasing at a volume that no single investigator can assimilate. The existence of this vast knowledge base creates the need for specialized computer databases to store and selectively sort the information. We have developed a manually curated database of the effects of hypertonicity on target proteins. Effects include changes in mRNA abundance and protein abundance, activity, phosphorylation state, binding, and cellular compartment. The biological information used in this database was derived from three research approaches: transcriptomic, proteomic, and reductionist (hypothesis‐driven). The data are presented in the form of grammatical triplets consisting of subject, verb phrase, and object. The purpose of this format is to allow the data to be read from left to right as an English sentence. It is readable either by humans or by computers using natural language processing algorithms. An example of a data entry reads “Hypertonicity increases activity of ABL1 in HEK293.” This database was created to provide access to a wealth of information on the effects of hypertonicity in a format that can be selectively sorted.

## Abstract (precis)

We have developed a manually curated database of the effects of hypertonicity on target proteins. Effects include changes in mRNA and protein abundance, activity, phosphorylation state, binding, and cellular compartment. This database was created to provide access to a wealth of information on the effects of hypertonicity in a format that can be selectively sorted.

## Introduction

The quantity of biological information continues to increase each year, vastly exceeding the ability of scientists to assimilate it into their knowledge stores. Consequently, there is a disconnection between the information available and the information that is actually utilized. To remedy this situation, databases have been developed to curate biological information, for example, STRING: functional protein association networks (http://string-db.org) and Ingenuity Pathway Analysis (http://www.ingenuity.com). Our experience is that these databases are too general to be useful in specialized areas such as renal physiology. Here, we have compiled a prototype database based on available information concerning osmoregulation in mammalian cells.

Specifically, we describe a new database containing a comprehensive list of hypertonicity‐induced effects on proteins or mRNA, the information for which has been culled from the biological literature. The data are presented in the form of grammatical triplets consisting of <subject><verb phrase><object>. Because the database is about hypertonicity effects, all the subject terms are “hypertonicity.” The verb phrases are actions such as “increases phosphorylation of” or “decreases abundance of.” The objects are given as official gene symbols corresponding to the affected proteins. This triplet structure is used to allow the database to be read either directly by humans or by computational parsing programs. The latter is necessary to facilitate computational network building. An example of a similar database on a different topic, namely vasopressin actions in the kidney, is offered by Sanghi et al. (2014).

## Methods

For our database, we used multiple sources. One source was the result of direct literature searching accomplished as part of the normal day‐to‐day research of the final two authors on this paper over the past two decades. The specific search terms were: “taurine transport” OR “sorbitol” OR “betaine” OR “glycerophosphocholine” OR “inositol” OR “hypertonicity” OR “hyperosmolality” OR “osmoregulation” OR “NTE” OR “TonEBP” OR “OREBP” OR “NFAT5” OR “gde2” OR “gdpd5” OR “osmostress” OR “osmotic stress” OR “smit.” In addition, we performed extensive additional PubMed searches prior to finalizing the database to insure that all relevant experiments in which “hypertonicity” was the independent variable were included. Search terms were: “osmotic,” “hyper,” “hypertonicity,” “osmostress,” “osmotic stress,” and “osmoregulation.” In each of our database entries the <subject> is “hypertonicity.” We included hypertonic effects due to elevated extracellular inorganic ions such as NaCl as well as organic solutes such as sorbitol and raffinose. Effects of urea are not included because most of its effects are due to its denaturing ability rather than effects on tonicity (Yancey et al. 1982). The list of database entries was supplemented by data from two proteomic data sets. The first was from a study of changes in protein abundance in the nucleus of HEK293 cells in response to hypertonicity (Li et al. 2012). The other was from a study of changes in phosphorylation of proteins in HEK293 cells in response to hypertonicity (Wang et al. 2014). To curate the data, we initially organized the details about each study in an electronic spreadsheet (*Excel,* Microsoft). We constructed each database entry to conform to English grammar syntax and to read from left to right as <subject><verb phrase><object><prepositional phrase><parenthetical information>. Next we used the spreadsheet to generate a Hyper Text Markup Language (HTML) file, as previously described in the Appendix of Sanghi et al. (2014). This was placed on a publicly accessible web server at https://helixweb.nih.gov/ESBL/Database/Osmo/. We provide a link to download a data submission form (https://helixweb.nih.gov/ESBL/Database/Osmo/Data_Submission_Form.xlsx) that can be filled out and submitted to the authors for inclusion in the online database.

The database was analyzed in part by mapping the gene symbol list (<objects>) to either Gene Ontology descriptors or Protein Domains using the program *Automated Bioinformatics Extractor* (ABE, http://helixweb.nih.gov/ESBL/ABE/). This program finds the appropriate Swiss‐Prot record corresponding to each gene symbol and extracts text strings corresponding to the desired type of data, which are transferred to a spreadsheet for collation. When a given gene symbol was represented more than once in the database, it was entered into ABE only once. Terms reported, such as “DNA binding” are standard *Gene Ontology Molecular Function* terms that are assigned by *Gene Ontology* curators.

## Results

**Figure 1.**
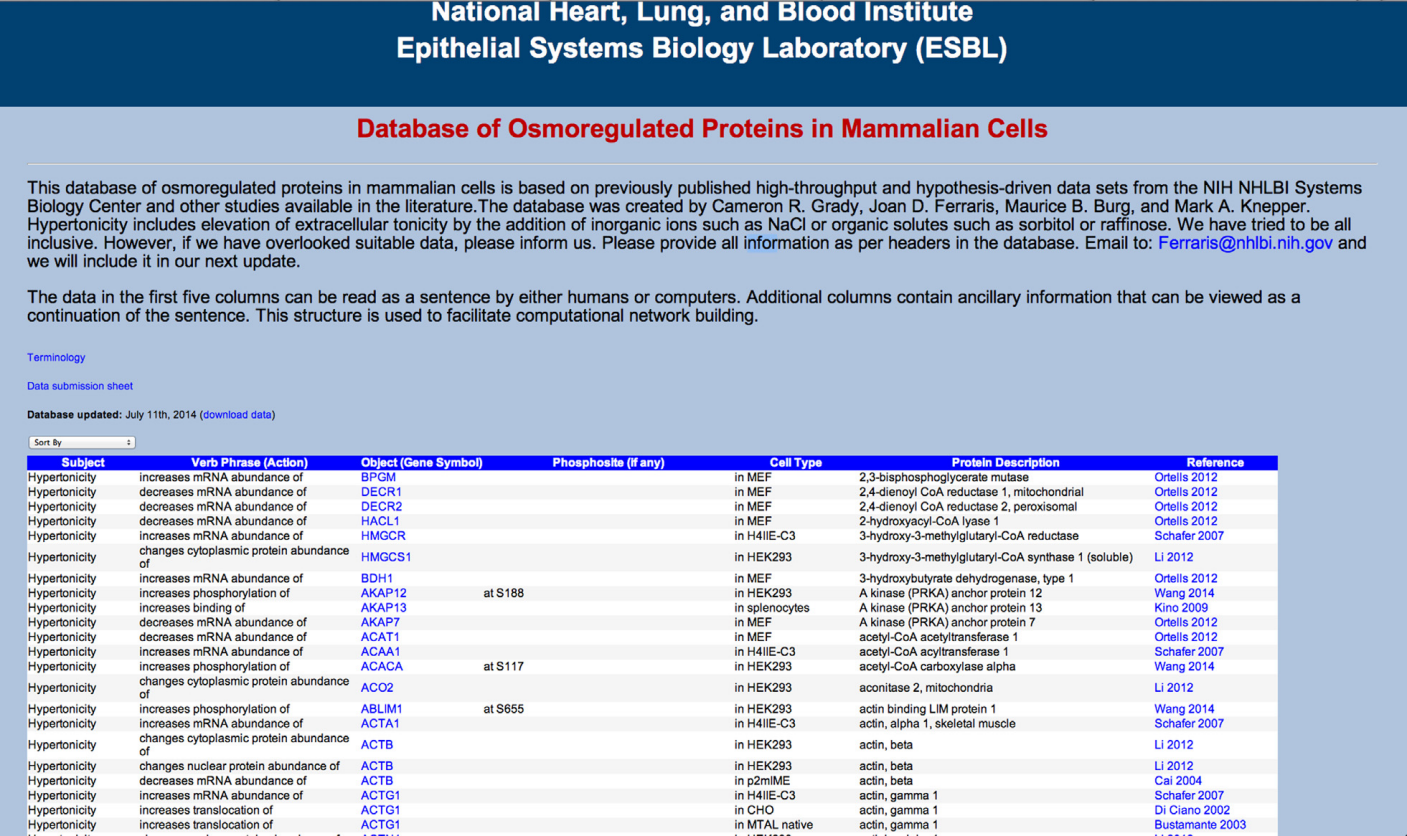
A screenshot image of the top of the webpage.

The *Database of Osmoregulated Proteins in Mammalian Cells* can be accessed at (https://helixweb.nih.gov/ESBL/Database/Osmo/). A screenshot image of the top of the webpage is shown in Figure 1. The first three columns represent the subject, verb phrase (action), and object (gene symbol) of each individual syntactical triplet. The three columns, together with the next two, form an English sentence when read from left to right. The last two columns indicate a description of the protein and the data source publication, which is hyperlinked to the appropriate PubMed entry. The specialized terminology is defined in a separate file that is viewable by placing the cursor on the word “Terminology.” The data may be downloaded into an electronic spreadsheet by clicking on “download data.” The database can be sorted in different ways using the dropdown list. The data on the webpage can be updated by sending a data submission form (https://helixweb.nih.gov/ESBL/Database/Osmo/Data_Submission_Form.xlsx) to the email address indicated on the website.

**Figure 2.**
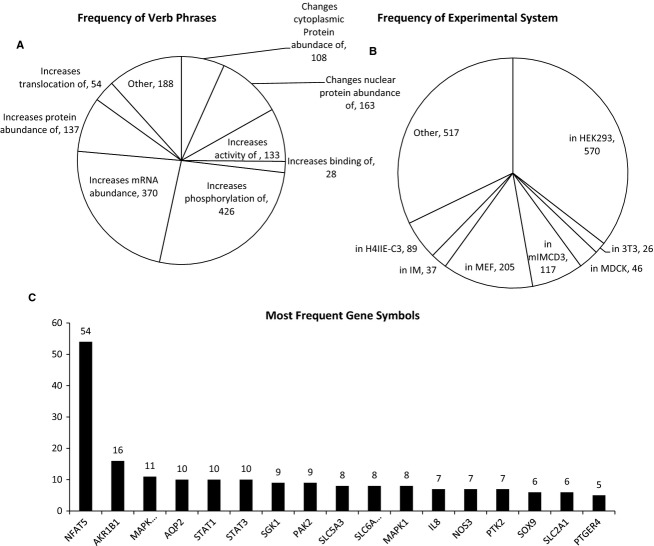
Characteristics of the *Database of Osmoregulated Proteins in Mammalian Cells*. (A) A pie chart showing the frequency of verb phrases or effects on target proteins such as changes in phosphorylation or abundance as found in the database. (B) A pie chart showing frequency of experimental system, often cell type, used in the studies cited in the database. (C) A bar graph of the most frequent target proteins, shown as gene symbols, found in the database.

Figure 2 describes some characteristics of the *Database of Osmoregulated Proteins in Mammalian Cells*. Figure 2A shows the frequency of each verb phrase. The most frequent entry is “increases phosphorylation of” with 426 entries. In Figure 2B, we show the frequency distribution of the experimental systems used. Figure 2C presents the frequency distribution of the gene symbols. NFAT5 (TonEBP/OREBP) was the most frequently referenced gene symbol by a wide margin. NFAT5 is an osmotically regulated transcription factor (Burg et al. 2007). The next most frequent were AKR1B1 (aldose reductase), MAPK14 (p38 kinase), and AQP2 (aquaporin‐2). (Note: The frequency of individual terms is given as a description of the database and does not necessarily equate with “importance”).

**Figure 3.**
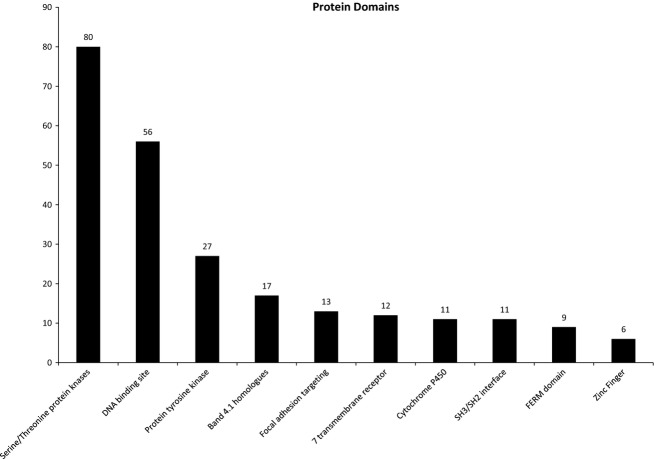
A bar graph of the most frequent protein domains found in target proteins on the database using *Automated Bioinformatics Extractor* (ABE, http://helixweb.nih.gov/ESBL/ABE/).

Figure 3 shows the most frequent protein domains found in proteins on the database. These data were extracted using the program *Automated Bioinformatics Extractor* (ABE, http://helixweb.nih.gov/ESBL/ABE/). This classification is informative because it gives a snapshot of the entire data set from the viewpoint of molecular interactions. The two most common domains are “serine/threonine protein kinases” and “DNA binding site.”

**Figure 4.**
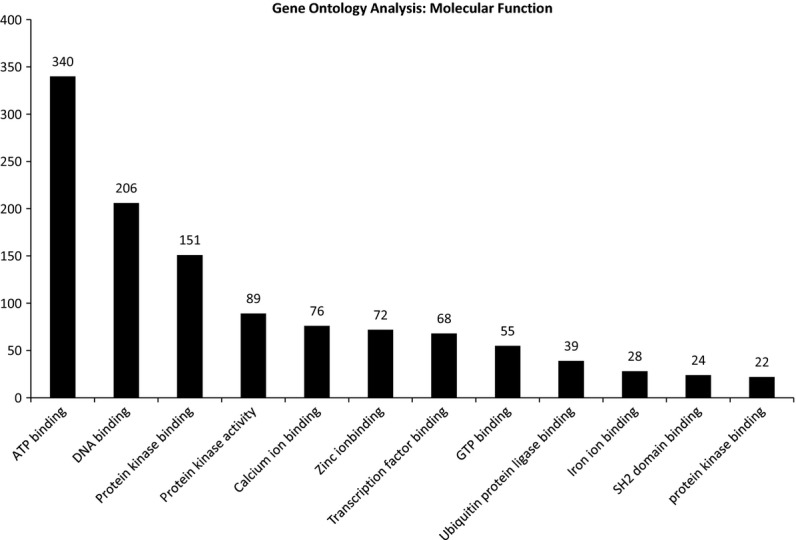
A bar graph of the most common molecular functions found for target proteins following Gene Ontology analysis using *Automated Bioinformatics Extractor* (ABE, http://helixweb.nih.gov/ESBL/ABE/).

Figure 4 shows the most frequent *Gene Ontology Molecular Function* terms found in proteins on the database. These data also were extracted using the program ABE. The most common terms were “ATP binding,” “DNA binding,” “protein kinase binding,” and “protein kinase activity.”

**Figure 5.**
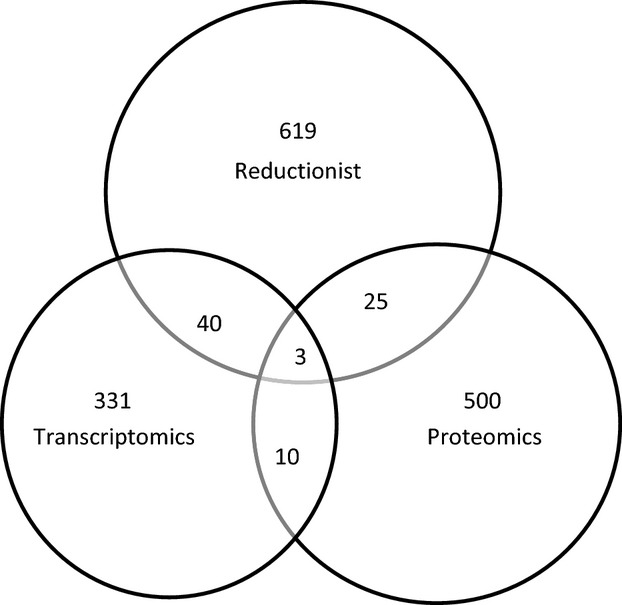
A Venn diagram showing the overlap in proteins among the three types of studies (transcriptomics, proteomics, and reductionist) that were incorporated into the database.

Each data entry was classified by the type of study: transcriptomics, proteomics, and reductionist. Figure 5 is a Venn diagram showing the number of proteins in each category. Only three proteins were found to be present in all three categories. These proteins were AKR1B1 (aldose reductase), HSPA1A (heat shock 70 kDa protein 1A), and ATP1A1 (ATPase, Na+/K+ transporting, alpha 1 polypeptide). Aldose reductase is an enzyme that converts cell glucose to the organic osmolyte sorbitol (Flynn 1982). Heat shock 70 kDa is an abundant cytosolic chaperone, buffering the cell from protein folding abnormalities in response to tonicity changes (Borkan and Gullans 2002). Na+/K+ ATPase is an ATP‐dependent transporter that moves sodium out of the cell and potassium into the cell, energizing cell volume regulation (Orlov et al. 2013).

## Discussion

Extraordinarily high extracellular osmolality is essential for the normal functioning of the renal medulla. Without elevated NaCl and urea concentrations, mammals could not concentrate urine and water balance would be compromised (Pannabecker 2013). Important questions previously addressed the effects of tonicity on the cells that comprise the renal medulla as well as the cells that pass through the medulla itself. Notably, high NaCl has major perturbing effects on cell function including cell death or apoptosis. However, cells also exhibit multiple mechanisms to adapt to hypertonicity. Overall, the effect of tonicity on cell function has been an active area of study for many years and a very large volume of literature has accumulated (Burg et al. 2007). Even in tissues not commonly considered to endure variation in tonicity, a modest hypertonic milieu may naturally occur. Such is the case for nucleus pulposus cells of the intervertebral disc (Tsai et al. 2007) and developing thymocytes (Trama et al. 2002).

Perhaps, in earlier days, an individual could keep track of and store all of the published data in their field. Then, it may have been possible to integrate all of the knowledge and form synthetic approaches to further investigations. More recently, with the advent of “omics‐” based approaches, the available data have proliferated hugely and we now have extraordinarily enhanced access to these data. At this point, the same task of gathering, assimilating, and synthesizing is beyond the capacity of the human brain. As an aid to investigators, we have developed the “Database of Osmoregulated Proteins in Mammalian Cells.” The database will be maintained online where it can be easily accessed, sorted, and viewed.

The “Database of Osmoregulated Proteins in Mammalian Cells” is a compilation of reported effects of hypertonicity on proteins and mRNAs. The format of the database is readable by humans directly or by computers (Evans and Rzhetsky 2011; Rebholz‐Schuhmann et al. 2012) using standard sentence‐parsing algorithms, for example, the Stanford Parser (http://nlp.stanford.edu/software/lex-parser.shtml). We constructed each database entry to conform to English grammar syntax and to read from left to right as <subject><verb phrase><object><prepositional phrase><parenthetical information>. The syntax allows a researcher to view a database entry and immediately have a basic understanding of information presented. There is no need to refer to the column header text as the information is in a simple English sentence. For example, one database entry reads “Hypertonicity decreases mRNA abundance of JUN in 3T3.” This is followed by the protein description “jun proto‐oncogene” and the appropriate reference “Xia 2007.” The official gene symbol is used as <object> and is linked to the NCBI gene page, whereas clicking on the reference links the user to the publication abstract.

In each of our database entries the <subject> is “hypertonicity.” The database includes approximately 1600 entries. Since tonicity can be bidirectional, the database also might be used to query what might occur if tonicity were decreased, in essence, changing the subject to hypotonicity. For example, finding an entry that states that “Hypertonicity increases phosphorylation of MAP3K3 at S526 in HEK 293” could lead to the hypothesis that hypotonicity decreases phosphorylation at that serine. A change in phosphorylation can be associated with a change in activity. For example, in a study examining effect of an siRNA library against all known phosphatases on NFAT5 activity, there were 57 siRNAs that changed NFAT5 transcriptional activity (Zhou et al. 2010). At high NaCl, 31 increased activity indicating that the phosphatase was inhibitory, implying that phosphorylation, presumably by a kinase, was stimulatory. In comparison, 16 decreased activity. The phosphorylation could be directly on NFAT5 or on a signaling element in its activation pathway. The database can be queried for proteins that have a desired attribute with respect to change in tonicity, as per the following scenario. An investigator identifies a consensus phosphorylation site in a protein of interest, mutates it so that it cannot be phosphorylated, and finds the mutation increases activity of the protein with exposure to elevated NaCl. The consensus database provides for multiple kinases that could phosphorylate at that site, but which one to test? Perusing our database could narrow the search by identifying kinases whose activity or phosphorylation changes with hypertonicity.

The <verb phrases> or actions that appear in the database are effects on change in protein abundance or translocation, change in cleavage or shedding, change in protein activity or binding, change in acetylation, phosphorylation or ubiquitination, change in mRNA abundance, and change in mRNA transcription rate.

The <objects> represented 1017 genes out of more than 21,000 genes in the mammalian genome. Importantly, we used the official gene symbol of the protein to represent <object>. Proteins commonly have redundant or ambiguous names and usage is not consistent. The use of official gene symbols largely avoids the ambiguity, inaccuracy, and redundancy inherent in protein nomenclature.

In addition to providing a venue for human users to readily access and assimilate the published information, the database is intended to facilitate automated data extraction. The database structure of <subject>, <verb phrase>, <object> is a triplet that a parser algorithm, using syntactical context, can extract from text to identify relationships among individual words (Evans and Rzhetsky 2011; Rebholz‐Schuhmann et al. 2012).

In summary, we have provided an expert‐curated database of osmoregulatory responses at a molecular level that can be mined for experimental design, systems‐biology type modeling, or as a shortcut to literature review. The format for data presentation is such that human readers or computers can interpret each data entry as a simple sentence, thereby facilitating data acquisition. It is hoped the experts throughout the field of physiology will record their own knowledge bases in a similar way to preserve the information and to facilitate large‐scale data integration with other data sets.

## Acknowledgments

C.R.G., M.A.K., M.B.B., and J.D.F. collected data, wrote, and reviewed the MS. The authors thank Dr. Chin‐Rang Yang for technical assistance.

## Conflict of Interest

The authors have no conflict of interest to declare.
